# Prediction of Final Rolling Temperature for TiAl Alloy Hot Rolling Based on Machine Learning

**DOI:** 10.3390/ma18071506

**Published:** 2025-03-27

**Authors:** Wei Lian, Fengshan Du, Qian Pei

**Affiliations:** 1 National Engineering Research Center for Equipment and Technology of Cold Strip Rolling, Yanshan University, Qinhuangdao 066004, China; lianwei21@outlook.com; 2 School of Information Science and Engineering, The Key Laboratory for Special Fiber and Fiber Sensor of Hebei Province, Yanshan University, Qinhuangdao 066004, China

**Keywords:** finishing rolling temperature, genetic algorithm, fuzzy theory, neural network, TiAl alloys

## Abstract

The final rolling temperature has a significant impact on the grain recrystallization and mechanical properties of rolled materials and is a key factor in the rolling process. With the development of the aerospace industry, higher requirements have been put forward for the quality of TiAl alloys. The suitable rolling temperature range of TiAl alloys is high and narrow, making it difficult to accurately control the final rolling temperature in real-time under the influence of environmental heat transfer and rolling heat. Finite element analysis can simulate the temperature fields, but takes a long time and is not suitable for online monitoring. Neural networks have the characteristic of fast response speeds and can be used for online control and rolling plan optimization. This article proposes a BP neural network prediction model (GABP) based on a genetic algorithm to predict the final rolling temperature. In order to determine the input parameters of the neural network, MATLAB was used to analyze the effects of various factors on the final rolling temperature. The prediction error of GABP is mainly concentrated at 0–1 °C. Compared with fuzzy neural networks (FNN), GABP has a higher prediction accuracy and can effectively predict the final rolling temperature of a TiAl alloy.

## 1. Introduction

The final rolling temperature has a significant impact on the recrystallization of grains and the mechanical properties of rolled parts. The final rolling temperature is influenced by the coupling of various rolling conditions, making it difficult to accurately solve. GABP is a new prediction model for predicting important parameters of rolling mills. A neural network model based on hot rolling can predict important parameters under many working conditions, such as changes in plate size, static and dynamic recrystallization kinetics, and the mechanical properties of rolled parts. Due to the inability to directly measure the final rolling temperature, most of them are based on numerical methods, such as developing prediction software [1]. However, neural networks have become a popular tool for analyzing metal processing problems due to their flexible learning abilities. Neural network models have been used to analyze metallurgical processes and predict rolling parameters, such as flow stress during rolling [2], tensile performance evaluation [3], prediction of strip flatness during cold continuous rolling [4], prediction of mechanical cooling systems [5], microstructure analysis [6], etc. Fitsum Bekele Tilahun proposed an overall optimization framework for introducing solar thermal systems (ISTS) design using machine learning methods and evaluated different performance indicators [7]. Jiang M et al. constructed an online accurate prediction model for the hot crown of rolling rolls during the hot rolling process using neural networks [8]. Lee JH et al. successfully predicted the thermal conductivity of concrete containing composition, temperature, and moisture content using a backpropagation neural network [9].

During the rolling process, neural networks are also widely used, with the prediction of rolling force being more common. Cheol YP et al. accurately predicted the rolling force and rolling exit thickness using an ML algorithm [10]. S.Rath et al. used variable learning rate and conjugate gradient technology to predict rolling force through a backpropagation neural network, with a prediction accuracy of 94% [11]. A.R. Shahani et al. successfully predicted rolling force during the aluminum alloy hot rolling process by combining finite element analysis with neural networks [12].

There are also some studies on temperature prediction. Wouter M. Geerdes et al. proposed a hybrid neural network model, which significantly improves the accuracy of temperature prediction compared to a single neural network [13]. Rakhoon H et al. proposed a prediction model that combines classical mechanical models with neural network models, improving the accuracy of predicting rolling force and temperature [14]. Hosein Alaei et al. used a combination of finite element models, mathematical analysis models, and neural network models to accurately predict the temperature and thermal expansion of rolling rolls [15]. Perttu L et al. used an adaptive neural network model to predict the rolling temperature of rough rolling mill rolled parts, with an average prediction error of 5.6 °C [16]. A large number of researchers have made outstanding contributions to the process of predicting rolling temperatures using neural networks [8,13,14,15]. Compared with traditional calculations, the above research greatly improves the prediction accuracy. The improvement of prediction accuracy provides important assurance for product quality control.

In response to the narrow range of suitable rolling temperatures and high requirements for the final rolling temperatures of TiAl alloys, this paper establishes a BP neural network prediction model based on genetic algorithm optimization for predicting the final rolling temperature of a TiAl alloy, further improving the prediction accuracy. The control of the final rolling temperature of a TiAl alloy is an important aspect of its quality control. The influence of various parameters on the final rolling temperature was analyzed using MATLAB software, and the input parameters of the neural network were determined. BP neural networks have strong nonlinearity and generalization ability. However, they also have drawbacks such as slow convergence speeds, multiple iteration steps, easy trapping in local minima, and poor global search ability. Therefore, a genetic algorithm is used to optimize the BP neural network and find a better search space in the analysis space. Then, the BP neural network is used to search for the optimal solution in a small search space, achieving the goal of quickly and effectively predicting the final rolling temperature.

## 2. Determination of Input Parameters for Neural Networks

### 2.1. Heat Conduction Model

The following is the heat flow equation for rolled strips during hot rolling:

(1) $$\int_{A}(\frac{\partial W_{i}}{\partial y}k\frac{\partial T}{\partial y}+\frac{\partial W_{i}}{\partial z}k\frac{\partial T}{\partial z})dA-\int_{A}W_{i}\rho c\frac{\partial T}{\partial \tau }dA+\int_{A}W_{i}\dot{q}dA-\int_{C}W_{i}q_{n}dS=0$$

In the formula, T represents the temperature, and ρ, c, and k represent the density, specific heat capacity, and thermal conductivity of the rolled plate, respectively. $W_{i}$ is a weighted function, q is the volumetric deformation heat rate outside the deformation zone equal to zero, and $q_{n}$ is the heat flux on the boundary surface.

The frictional heat flux $q_{fric}$ between the rolling plate and the rolling roller is represented as follows:

(2) $$q_{fric}=\sigma_{s}A_{c}\left|\Delta \nu \right|\delta \tau$$

In the formula, $\left|\nu \right|$ and τ represent the relative velocity and time step, $A_{c}$ represents the contact area, and $\sigma_{s}$ represents the frictional stress.

In the deformation zone, there are the following boundary conditions:

(3) $$\begin{array}{c} -k\frac{\partial T}{\partial z}=h_{con}(T-T_{R})-q_{fric}-k\frac{\partial T}{\partial z}\left|\begin{array}{c} z=0 \end{array}\right.=0\\ -k\frac{\partial T}{\partial y}=h_{\infty }(T-T_{\infty })+\sigma \varepsilon (T^{4}-T_{\infty }^{4})-k\frac{\partial T}{\partial y}\left|\begin{array}{c} y=0 \end{array}\right.=0 \end{array}$$

In the above formula, $T_{R}$ represents the surface temperature of the work roller, $T_{\infty }$ represents the air temperature, and $h_{con}$ is the interfacial heat transfer coefficient based on different temperatures.

The convective heat transfer equation is as follows:

(4) ϕ=AhΔt

In the above formula, A represents the surface area, h represents the convection heat transfer coefficient, and Δt represents the overall temperature difference.

### 2.2. Analysis of the Influence of Various Factors on the Final Rolling Temperature

**Figure 1:**
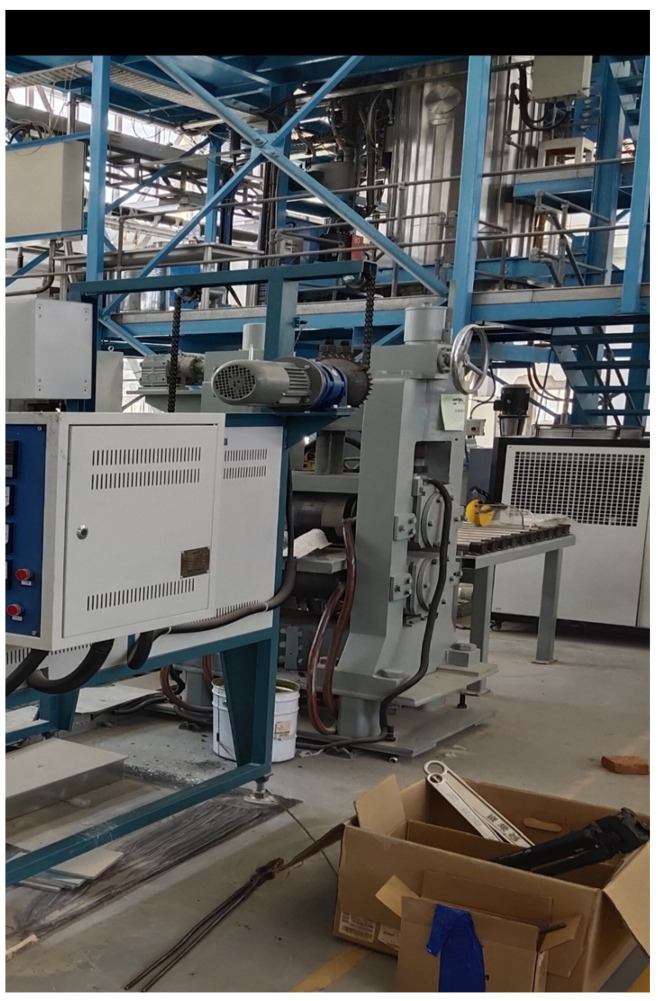
Experimental rolling mill.

**Figure 2:**
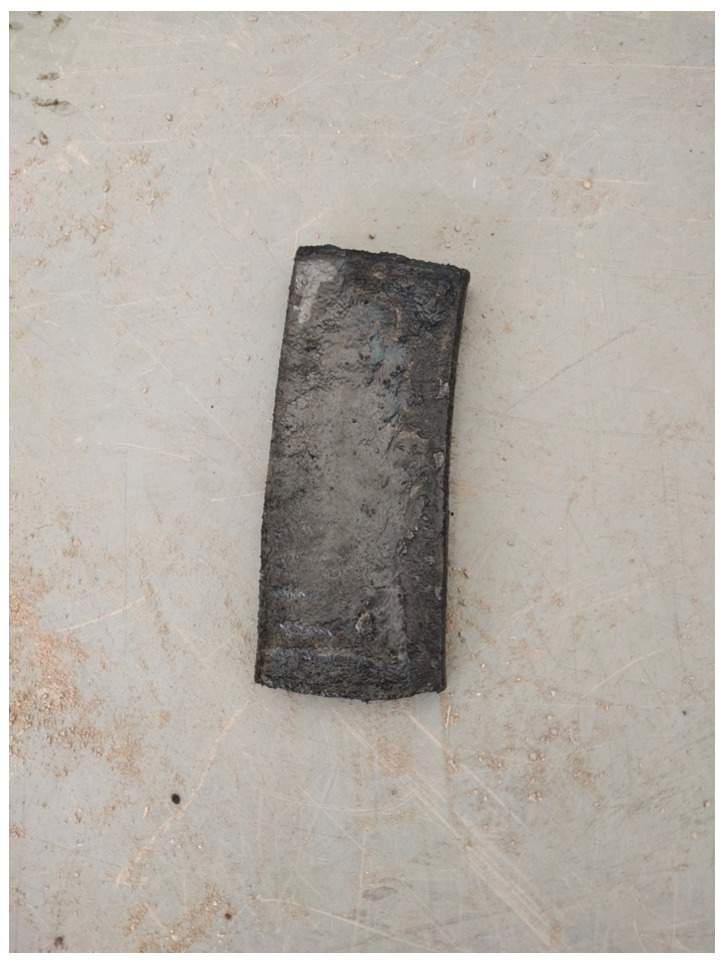
Experimental piece.

Utilizing MATLAB 2018 software, this study systematically investigated the effects of rolling speed, reduction rate, initial rolling temperature, and roller-workpiece friction coefficient on the final rolling temperature. The research aimed to elucidate the complex interdependencies among these critical rolling parameters and their cumulative influence on thermomechanical behavior during the rolling process. The experimental equipment is an electromagnetic rolling mill, as shown in Figure 1. This rolling mill is a two roll rolling mill, which can compensate for the heating of the rolling mill rolls through electromagnetic heating technology to achieve a stable rolling mill temperature within a certain range. Figure 2 shows the rolled material. Due to the narrow temperature range suitable for TiAl alloy rolling, an isothermal rolling process is required.

**Figure 3:**
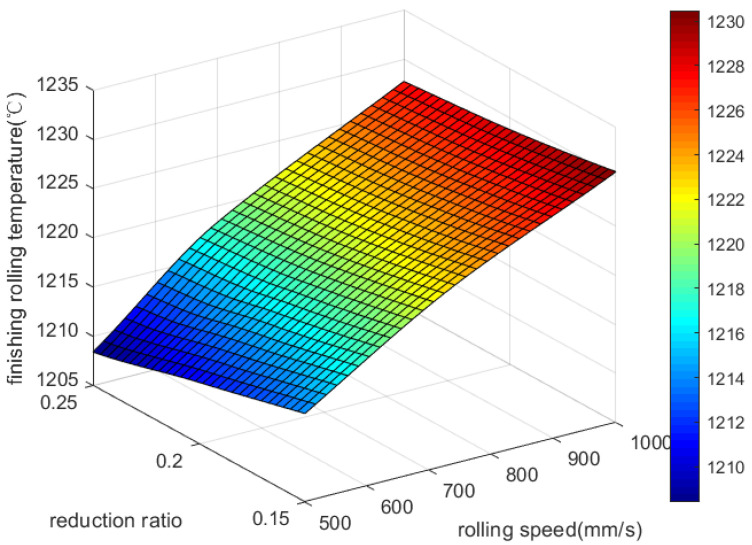
Effect of rolling speed and reduction rate on final rolling temperature.

It can be seen from Figure 3 that the final rolling temperature increases as the rolling speed increases. The final rolling temperature is more strongly influenced by the rolling speed. Increasing the rolling speed can shorten the rolling time, reduce the heat dissipation of the TiAl alloy during the rolling process, and increase the final rolling temperature. The final rolling temperature decreases when the reduction rate increases, mainly due to the increase in reduction rate. Rolling time increases significantly as well, and a decrease in the final rolling temperature results from a greater heat exchange than deformation heat.

**Figure 4:**
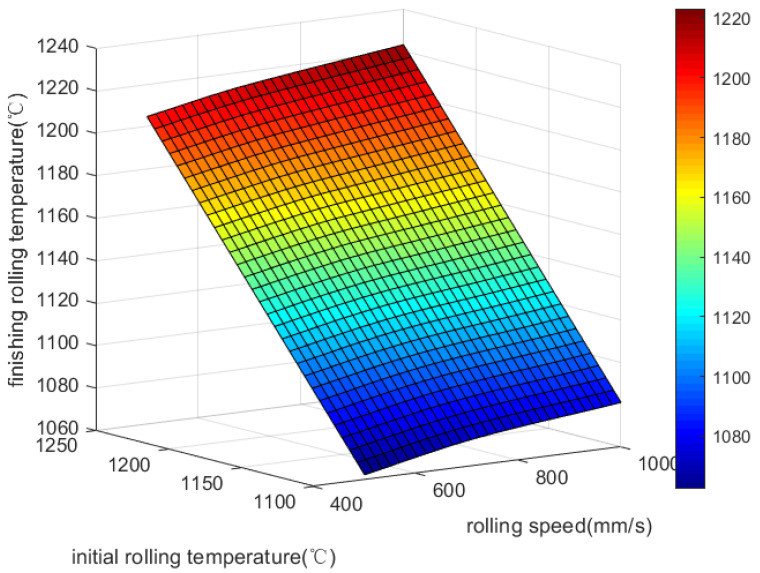
Effect of rolling speed and initial rolling temperature on final rolling temperature.

Figure 4 reveals that a gradual increase in rolling speed and initial rolling temperature leads to a gradual increase in rolling temperature. The final rolling temperature is significantly influenced by the rolling speed, but the inlet temperature determines the upper limit directly, so the influence of the inlet temperature on the finishing rolling temperature is more significant.

**Figure 5:**
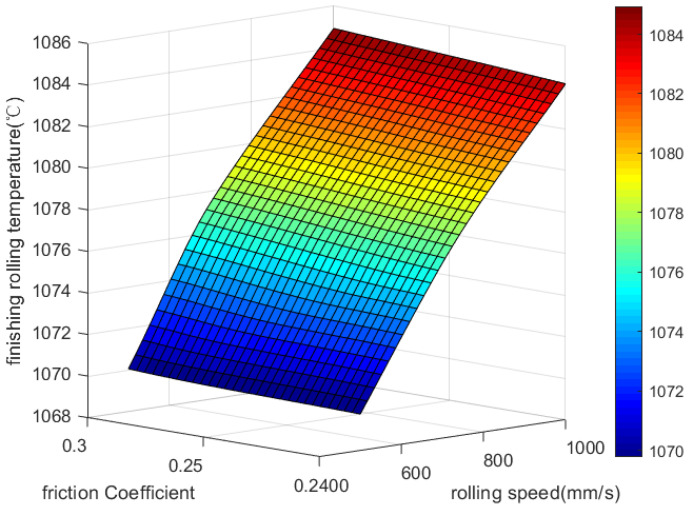
Effect of rolling speed and friction coefficient on final rolling temperature.

Figure 5 clearly illustrates that the final rolling temperature is less affected by the friction coefficient between the rolling roller and rolling plate than it is by rolling speed or rolling temperature. This is mainly due to the fact that no significant slip occurs during the rolling process, resulting in minimal frictional heating. As a result, the friction coefficient has a negligible effect on the final rolling temperature.

**Figure 6:**
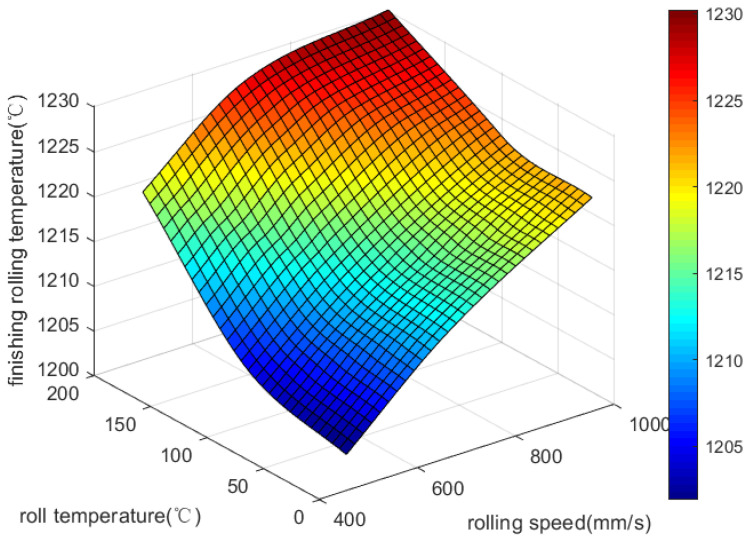
Effect of rolling speed and roll temperature on final rolling temperature.

Final rolling temperatures rise with increasing roller temperatures from 20 °C to 200 °C in Figure 6. When there is a decrease in the temperature difference between the rolling plate and rolling roller, the heat exchange between them decreases. A roller’s temperature impacts the final rolling temperature in a similar way to the speed at which it is rolled.

Ultimately, rolling temperature is the least affected by the friction coefficient between the roller and the workpiece. Because directly ignoring the friction coefficient can lead to a significant increase in the prediction error, a prediction is made by controlling the weight of the friction coefficient.

## 3. Development of Artificial Neural Network Models

### 3.1. Structure and Principles of Neural Network Models

**Figure 7:**
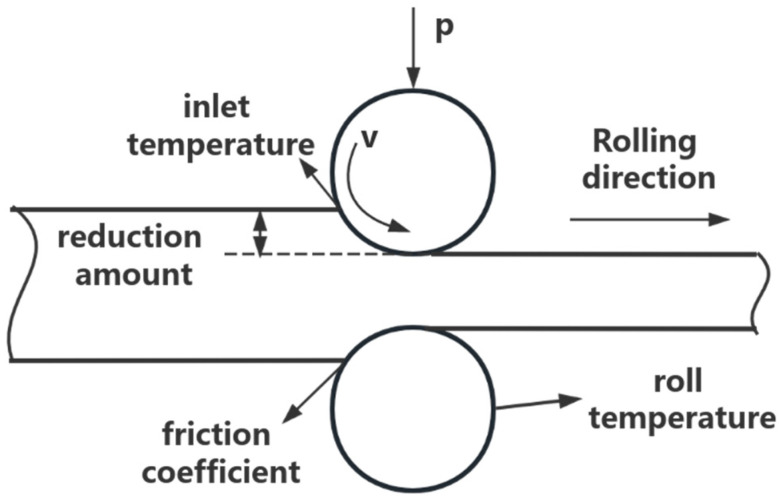
The rolling process.

**Figure 8:**
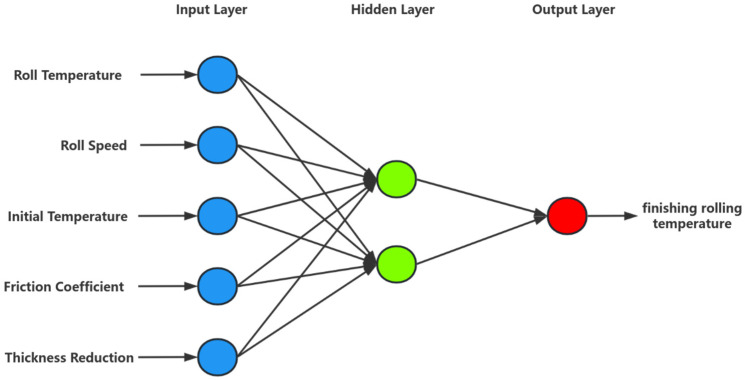
Neural network structure.

**Figure 9:**
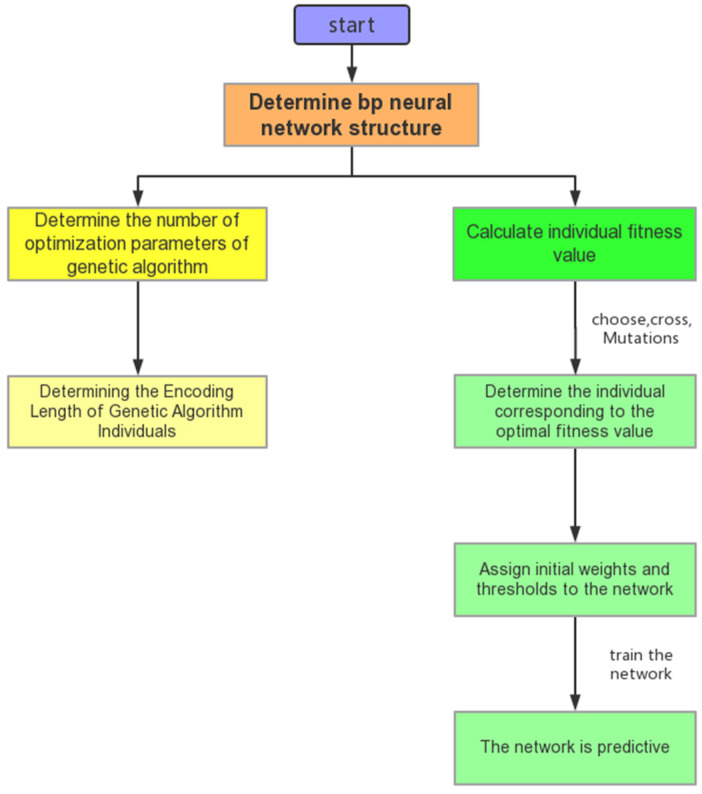
Optimization principle of genetic algorithm.

This article establishes a GABP model for predicting the final rolling temperature. Select the rolling temperature, rolling roller temperature, rolling speed, reduction rate, and friction coefficient as input parameters. The rolling process is shown in Figure 7. The neural network structure is shown in Figure 8. The principle of GABP is shown in Figure 9.

### 3.2. Neural Network Training and Testing Datasets

**Figure 10:**
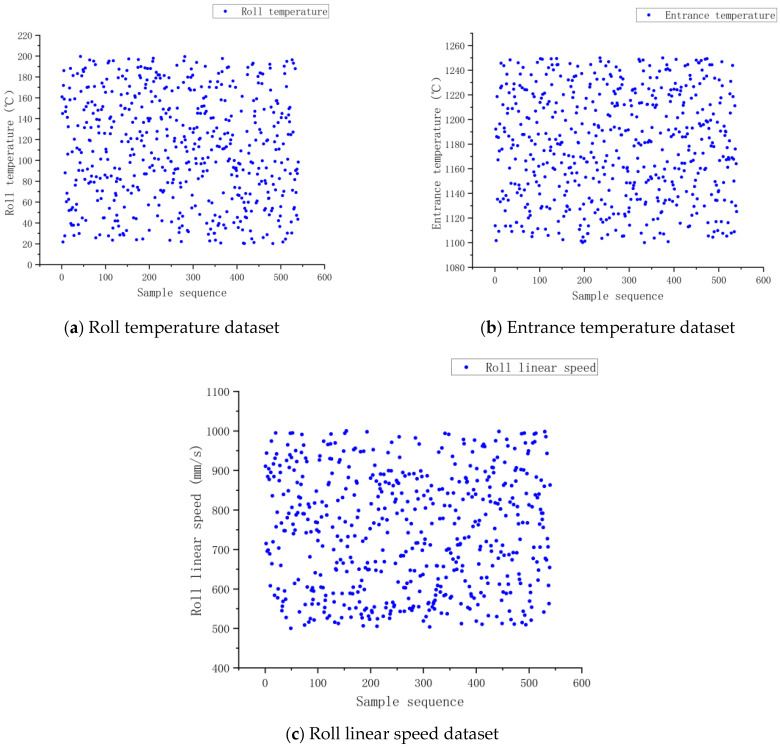
(**a**–**c**) Input parameter dataset.

**Figure 11:**
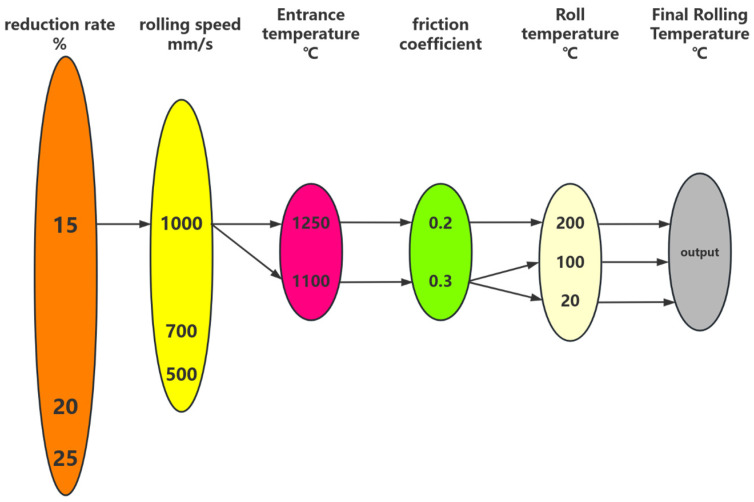
Principles for selecting test sets.

Figure 10 shows the dataset of input parameters for the neural network. The dataset includes 540 sets of data, of which 150 sets were used for testing. The test set was selected based on typical working conditions, as shown in Figure 11. Rolling mill bounce is present during the rolling process, but the amount of bounce is very small, and its impact can be considered in the impact of rolling speed on the final rolling temperature. The reduction rate in the input parameters can be represented by the preset roll spacing corresponding to the reduction rates, which were 15%, 20%, and 25%, respectively.

### 3.3. Choosing the Best Network Topology

Due to the strong modeling ability of BP neural networks, there are currently many mature backpropagation training algorithms, such as the gradient regression algorithm, quasi-Newton optimization algorithm, conjugate gradient algorithm, random approximation algorithm, Levenburg–MarQuardt optimization algorithm, etc. The hidden layer and output layer of the neural network adopted Tansig-type and Purelin-type transfer functions, respectively, and Trainlm was selected as the training function.

**Figure 12:**
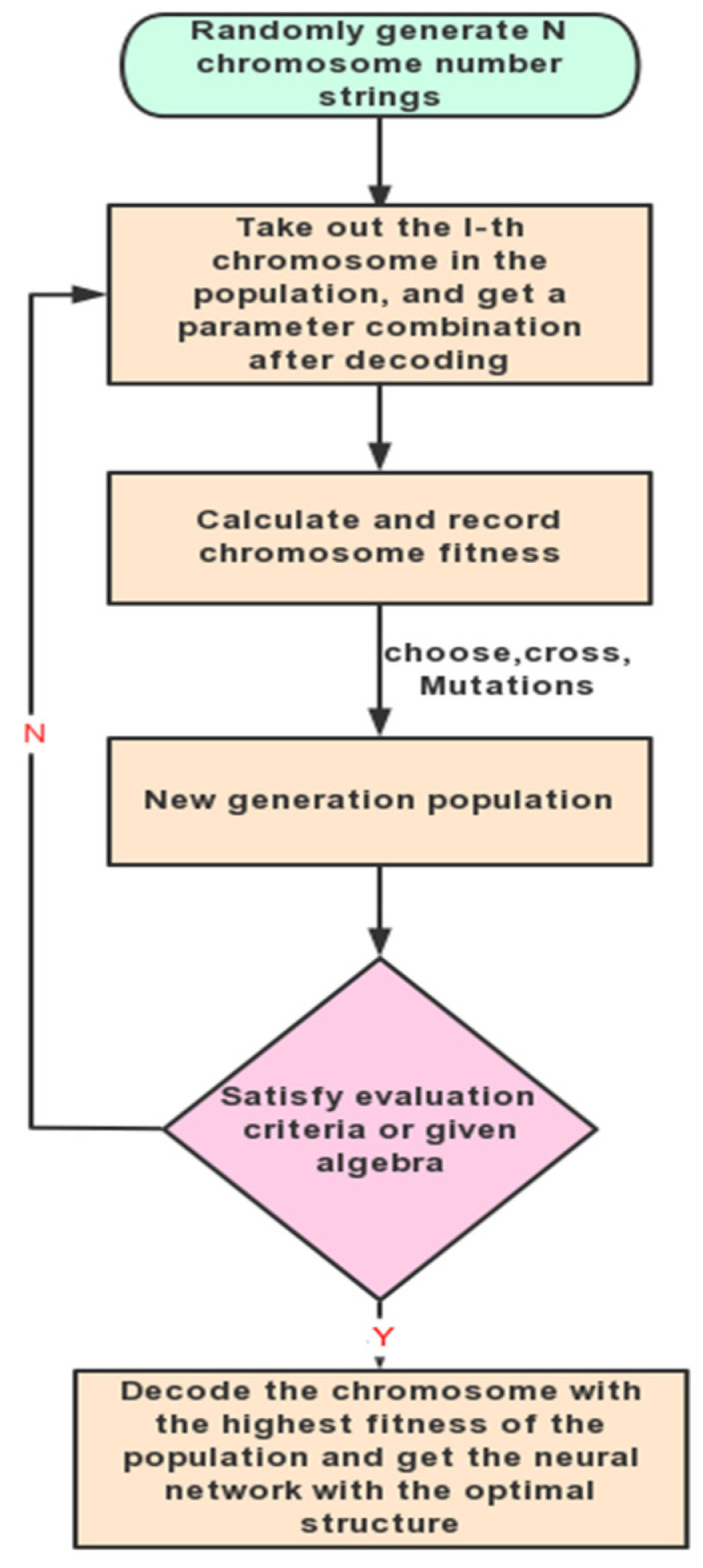
Genetic algorithm optimization process.

In order to improve the accuracy of the network’s predictions, we used genetic algorithms to optimize the network’s learning and structural design. By using global optimization and the implicit parallelism of genetic algorithms, the optimization speed of weight coefficients can be improved. The basic process of network optimization is shown in Figure 12.

Before transferring the optimal weights and thresholds to the BP neural network, it is usually necessary to normalize the input and target data to ensure that they fall within a specific range. Scale all input and target data within the range of [0, 1] using the following normalization equation:

(5) $$y=\frac{\left(y_{\max}-y_{\min})\times (x-x_{\min}\right)}{x_{\max}-x_{\min}}+y_{\min}$$

In the equation, $y_{\max}$ and $y_{\min}$ are 1 and 0 respectively, $x_{\max}$ and $x_{\min}$ are the maximum and minimum values in the original data, and x and y are the input and normalized data, respectively.

One of the most important tasks in artificial neural networks is to determine the number of hidden layers and neurons. Generally, trial-and-error methods are used. This study obtained the optimal network structure by attempting different numbers of hidden layers and neurons. The experiment starts with a hidden layer of one neuron, and the performance of each network is evaluated by the sum of errors (e). Take the minimum value of e to obtain a neural network with optimal generalization performance. We have tried many different network structures and calculated their e values. In a network with one hidden layer and two hidden neurons, the predicted e-value of the final rolling temperature is 192.3645 °C. Due to the 150 sets of data in the test set, the average error of each set was only 1.28 °C. Based on this, the optimal architecture for constructing an artificial neural network is 5-2-1, which represents the number of neurons in the input layer, hidden layer, and output layer, respectively.

## 4. Analysis and Exploration of Neural Network Models

**Figure 13:**
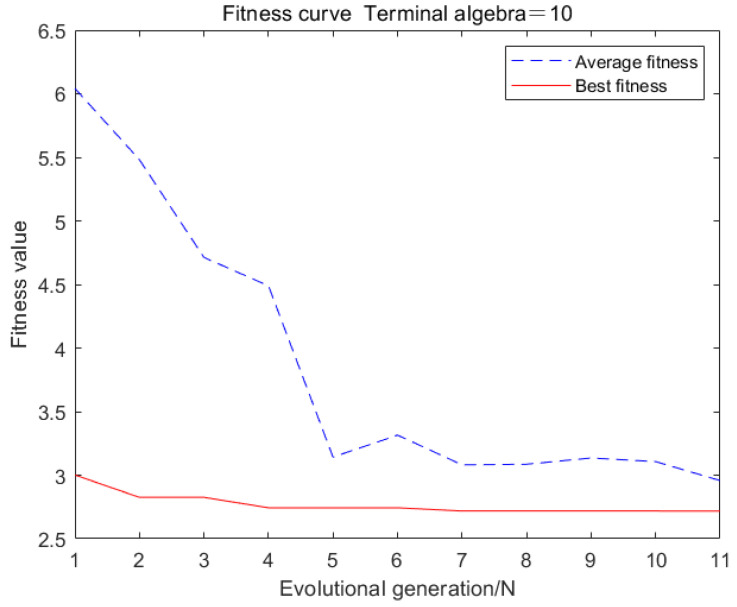
Fitness curve of genetic neural network.

**Figure 14:**
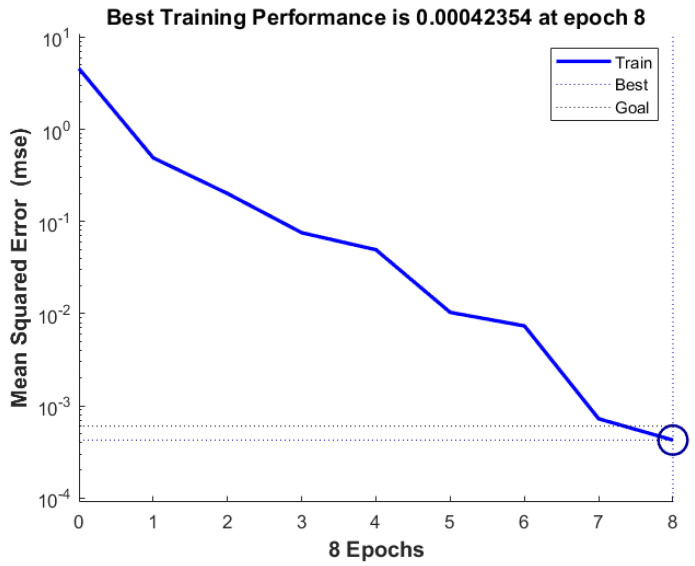
Genetic neural network prediction mean square error.

The neural network model established in this article verifies its prediction accuracy and feasibility by predicting 150 sets of final rolling temperature test sets. The fitness curve and the mean square error of the training data are shown in Figure 13 and Figure 14, respectively.

From Figure 13, it can be seen that the average fitness value is close to the optimal fitness value, indicating that each individual in the population is around the optimal solution and has good convergence.

**Figure 15:**
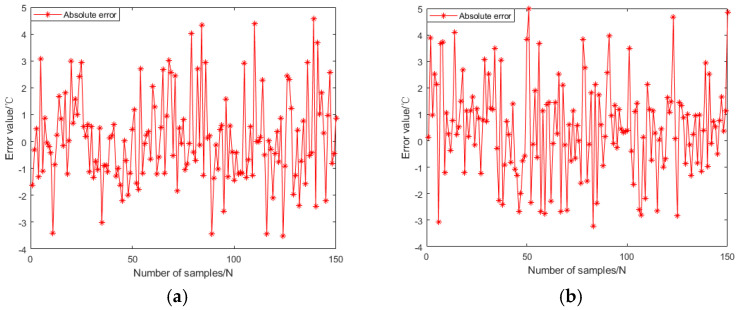
Absolute error of neural network prediction. (**a**) Genetic algorithm optimization of BP neural network; (**b**) Fuzzy algorithm optimization of BP neural network.

**Figure 16:**
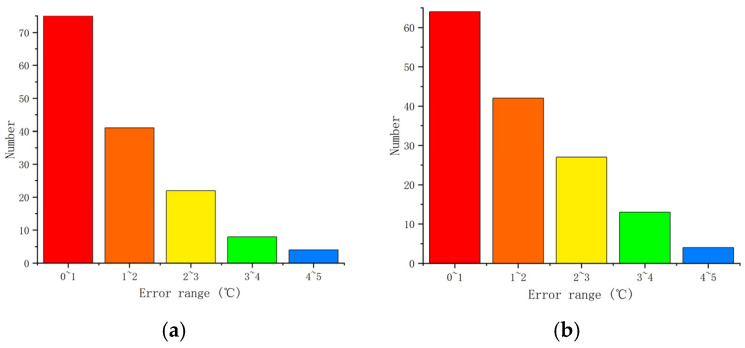
Absolute error distribution of neural network prediction. (**a**) Genetic algorithm optimization of BP neural network; (**b**) Fuzzy algorithm optimization of BP neural network.

**Figure 17:**
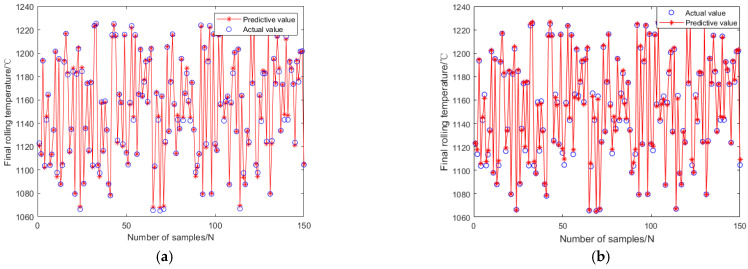
Neural network prediction effect chart. (**a**) Genetic algorithm optimization of BP neural network; (**b**) Fuzzy algorithm optimization of BP neural network.

From Figure 14, it can be seen that as the algebra increases, the mean square error of the training data gradually approaches 0.00042354. In addition, the absolute errors and distributions of genetic neural networks and fuzzy neural networks are shown in Figure 15 and Figure 16. The predictive performance of the two models is shown in Figure 17.

In Figure 15, it can be seen that the maximum prediction error of GABP is only 8.8 °C, while the maximum prediction error of FNNs is 10.9 °C. From Figure 16, it can be seen that the absolute error of GABP prediction is mainly concentrated at 0–1 °C, and there are only six groups of FNNs within this range.

As shown in Figure 17, the GABP prediction results are in good agreement with the test set, with a correlation coefficient of 0.9993. The results indicate that the GABP prediction model has high predictive ability for the final rolling temperature of TiAl alloys.

## 5. Conclusions

In order to achieve precise prediction of the final rolling temperature of a TiAl alloy during hot rolling, a novel Genetic Algorithm-Back Propagation (GABP) neural network model was developed. The selection of input parameters for the neural network was meticulously conducted using Matlab software, ensuring that the model incorporates the most relevant variables influencing the rolling process. The architecture of the BP neural network, particularly the number of neurons in the hidden layer, was optimized through a trial-and-error approach to achieve the best predictive performance. Furthermore, a genetic algorithm was employed to optimize the weights and thresholds of the neural network, thereby enhancing its generalization capability and predictive accuracy.

The validation results demonstrate that the GABP model exhibits superior performance in predicting the final rolling temperature of TiAl alloys under diverse hot rolling conditions. This model can be effectively utilized for online monitoring of the rolling process, providing real-time feedback to ensure process stability and product quality. Compared with the conventional FNN, the GABP model, as developed in this study, exhibits enhanced accuracy in predicting the final rolling temperature of a TiAl alloy based on actual rolling conditions. The model’s ability to precisely capture the final rolling temperature at each point of the final product is crucial to maintaining the desired microstructure and mechanical properties of TiAl alloys. This research underscores the significance of integrating advanced optimization techniques with neural network models to address complex industrial problems, thereby contributing to the improvement of TiAl alloy quality control and process optimization.

## Data Availability

The data presented in this study are available on request from the corresponding author. The data are not publicly available due to privacy restrictions.
